# Supplementary material for: Validation of an Immunoassay for Anti-thymidine Phosphorylase Antibodies in Patients with MNGIE Treated with Enzyme Replacement Therapy
Source: Mol Ther Methods Clin Dev. 2018 Aug 28;11:1–8. doi: 10.1016/j.omtm.2018.08.007 (PMC6170929; doi:10.1016/j.omtm.2018.08.007)
Supplement: Document S1. Figure S1 and Tables S1 and S2 [file mmc1.pdf]

OMTM, Volume *11*

Supplemental Information

Validation of an Immunoassay for Anti-thymidine Phosphorylase Antibodies in Patients with MNGIE Treated with Enzyme Replacement Therapy

Michelle Levene, Dario Pacitti, Charlotte Gasson, Jamie Hall, Marcia Sellos-Moura, and Bridget E. Bax

Supplemental Figure 1. Screening cut-point. To establish the screening cut-point, 51 individual control serum samples were analysed in duplicate by two analysts over three plates on three days. Significant differences were observed between means for analyst, day, plate, analyst*plate, analyst*day and analyst*day*plate interactions (p< 0.001) and variances (p<0.001). Data is expressed as mean RLU ± SD.

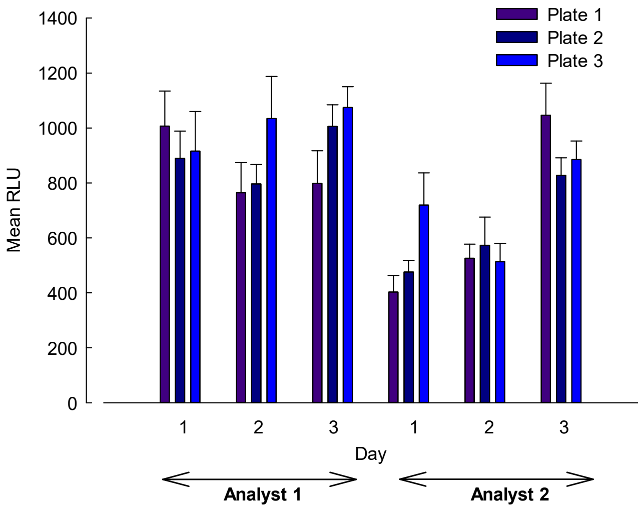

Supplemental Table 1. Comparison of instrument responses to negative control and disease state matrices.

Serum sample	Instrument response (RLU[a])	
	**Mean ± SD**	**CV (%)**
Disease (n=5)	1169 ± 388	33.2
Healthy control (n=10)	1301 ± 141	10.9

[a] RLU; relative light unit

Supplemental Table 2. Sensitivity analysis for Analyst 1 for each of 9 plates.

Plate	Mean RLU[a] Negative control	Screening cut point[b] (RLU)	Log sensitivity level
1	1037.0	1165.6	1.171
2	981.0	1109.6	2.233
3	905.0	1033.6	2.422
4	920.5	1049.1	3.114
5	818.0	946.6	2.472
6	810.5	939.1	2.243
7	987.5	1116.1	1.803
8	1107.0	1235.6	2.119
9	875.5	1004.1	2.431
**Log mean sensitivity level + SD**			2.223 ± 0.528
**Log mean sensitivity Upper 95% CI[c]**			2.551
**Mean sensitivity Upper 95% CI**			355.53

[a] RLU; relative light unit

[b]Screening cut point = Negative control mean + Correction Factor (128.6)

[c] Upper log mean sensitivity =Mean + (SD/√n)*t
