# Supplementary material for: Validation of an Immunoassay for Anti-thymidine Phosphorylase Antibodies in Patients with MNGIE Treated with Enzyme Replacement Therapy
Source: Mol Ther Methods Clin Dev. 2018 Aug 28;11:1–8. doi: 10.1016/j.omtm.2018.08.007 (PMC6170929; doi:10.1016/j.omtm.2018.08.007)
Supplement: Document S2. Article plus Supplemental Information [file mmc2.pdf]

Molecular Therapy
Methods & Clinical Development
Original Article

Validation of an Immunoassay for Anti-thymidine Phosphorylase Antibodies in Patients with MNGIE Treated with Enzyme Replacement Therapy

Michelle Levene,[1] Dario Pacitti,[1] Charlotte Gasson,[2] Jamie Hall,[2] Marcia Sellos-Moura,[3] and Bridget E. Bax[1]

[1]Molecular & Clinical Sciences Research Institute, St. George's, University of London, London, UK; [2]Biomarker, Bioanalysis and Clinical Sciences, Envigo CRS, Cambridgeshire, UK; [3]Orphan Technologies, Zuercherstrasse 19, Rapperswil, Switzerland

Erythrocyte encapsulated thymidine phosphorylase is recombinant *Escherichia coli* thymidine phosphorylase encapsulated within human autologous erythrocytes and is under development as an enzyme replacement therapy for the ultra-rare inherited metabolic disorder mitochondrial neurogastrointestinal encephalomyopathy. This study describes the method validation of a two-step bridging electrochemiluminescence immunoassay for the detection of anti-thymidine phosphorylase antibodies in human serum according to current industry practice and regulatory guidelines. The analytical method was assessed for screening cut point, specificity, selectivity, precision, prozone effect, drug tolerance, and stability. Key findings were a correction factor of 129 relative light units for the cut-point determination; a specificity cut point of 93% inhibition; confirmed intra-assay and inter-assay precision; assay sensitivity of 356 ng/mL; no matrix or prozone effects up to 25,900 ng/mL; a drug tolerance of 156 ng/mL; and stability at room temperature for 24 hr and up to five freeze-thaws. Immunogenicity evaluations of serum from three patients who received erythrocyte encapsulated thymidine phosphorylase under a compassionate treatment program showed specific anti-thymidine phosphorylase antibodies in one patient. To conclude, a sensitive, specific, and selective immunoassay has been validated for the measurement of anti-thymidine phosphorylase antibodies; this will be utilized in a phase II pivotal clinical trial of erythrocyte encapsulated thymidine phosphorylase.

INTRODUCTION

Enzyme replacement therapies are typically applied to the treatment of individuals with inherited enzyme deficiency disorders, whereby the deficient enzyme is replaced by regular infusions of the normal counterpart, with the aim of decelerating the disease progression process. Current licensed preparations are either purified from natural human or animal sources, or produced by recombinant technologies, and thus have the potential to induce undesirable immune responses. Clinical experience has shown that the development of anti-enzyme antibodies is a common occurrence, with many of the approved enzyme replacement therapies exhibiting immunogenicity rates of 51%–100%.[1,2] Clinical complications of immunogenic reactions include the modification of therapeutic efficacy and acute infusion reactions, such as anaphylaxis. Appropriately, the appraisal of anti-enzyme antibody formation is a crucial component of the clinical development program and is specifically relevant during the evaluation of the enzyme's efficacy and safety profile. There is thus a regulatory expectation that a valid, sensitive, specific, and selective immunoassay is developed for measuring enzyme-specific antibody responses.[3,4]

Erythrocyte encapsulated thymidine phosphorylase (EETP) is under development as an enzyme replacement therapy for the rare metabolic disorder mitochondrial neurogastrointestinal encephalomyopathy, abbreviated to MNGIE.[5–7] The disease is caused by mutations in the nuclear *TYMP* gene encoding for the enzyme thymidine phosphorylase (TP), leading to elevated concentrations of thymidine and deoxyuridine in cellular and extra-cellular compartments, and ultimately mitochondrial failure due to progressive accumulation of mtDNA defects and mtDNA depletion.[8–12] Clinically, MNGIE manifests as leukoencephalopathy, ptosis and ophthalmoplegia, peripheral polyneuropathy, and enteric neuromyopathy, causing severe gastrointestinal dysmotility with cachexia.[13] The disorder invariably leads to death at an average age of 37.6 years.

EETP is produced by encapsulating recombinant *Escherichia coli* (*E. coli*) TP within autologous erythrocytes *ex vitro*; the loaded cells are then infused into the patient. The rationale for this approach is based on thymidine and deoxyuridine diffusing across the erythrocyte membrane via nucleoside transporters into the cell where the encapsulated enzyme catalyzes their metabolism to the normal products. The administration of EETP under a compassionate treatment program has shown a sustained reduction or elimination of plasma thymidine and deoxyuridine concentrations, translating into clinical improvement.[5,6,14,15] EETP therapy has the advantage

Received 17 July 2018; accepted 21 August 2018;
https://doi.org/10.1016/j.omtm.2018.08.007.

Correspondence: Bridget E. Bax, Molecular & Clinical Sciences Research Institute, St George's, University of London, London SW17 0RE, UK.
E-mail: bebax@sgul.ac.uk

This is an open access article under the CC BY license (http://creativecommons.org/licenses/by/4.0/).

Table 1. Summary of Key Validation Parameters for the Assessment of Anti-TP Antibodies in Human Serum

Validation Parameter	Results
Positive control standard range	2.44–10,000 ng/mL
Correction factor for cut-point calculation	129 RLUs
Screening cut point	floating cut point
Specificity cut point	93.0%
Assay sensitivity	356 ng/mL
Intra-assay Performance	Precision, CV (%)
Negative control	14.5
Low positive control	11.1
High positive control	1.0
Inter-assay Performance	Mean Precision, CV (%)
Negative control	43.3
Low positive control	40.6
High positive control	30.5
Assay drift	not present
Minimum required dilution (MRD)	1 in 10
Selectivity (matrix effects)	not present
Prozone	not present up to 25,900 ng/mL
Drug tolerance	tolerant up to 156 ng/mL
Confirmatory drug concentration	12,500 ng/mL
Stability	
Room temperature	up to 24 hr
Freeze-thaw	up to five freeze-thaw cycles

RLU, relative light unit.

Table 2. Screening Cut-Point Determination

Parameter	RLUs	
	First Iteration	Second Iteration
Mean	797.9	914.3
N	36	18
SD	61.2	92.6
Cut point	898.5	1,066.6
Mean of negative controls	814.2	938
Correction factor	–	128.6

The cut point was determined using 51 individual lots of serum, analyzed in duplicate, by 2 analysts, over 3 plates, on 3 days. The cut point was calculated as the RLUs + 1.645*SD. The first iteration represents data analyzed from analysts 1 and 2. Data from analyst 2 were removed for the second iteration.

of prolonging the circulatory half-life of the enzyme and potentially minimizing the immunogenic reactions, which are frequently observed in enzyme replacement therapies administered by the conventional route.

We describe here the validation of a two-step immunoassay method for the detection of anti-TP antibodies in human serum for supporting a phase II pivotal clinical trial of EETP. The analytical method was assessed for screening cut point, specificity, intra-assay and inter-assay precision, sensitivity, selectivity, drug tolerance, prozone effect, and stability.

RESULTS

The key results from this validation study are presented in Table 1.

Disease State Matrix

Of the seven disease matrix samples from untreated patients that were screened, five were negative for anti-TP antibodies. The difference in the mean instrument responses between the patient and normal matrix samples was 10.1%; this was not considered to be significant (see Table S1), indicating that the same cut point can be applied (see below).

Screening and Specificity Cut Point

The signal distribution for the 51 negative control (NC) samples was normally distributed ($p > 0.05$) with no outliers. The validation cut point was calculated to be 898.5 relative light units (RLUs) (see Table 2, first iteration).

Statistically significant differences were evident between the means for analyst, day, plate, analyst by plate, analyst by day, and analyst by day by plate interactions ($p < 0.001$) and also the variances ($p < 0.001$) indicating a dynamic screening cut point (see Figure S1). Each analyst was analyzed separately to determine the source of these differences. For analyst 1, there were significant differences between the means for day, plate, and their interaction, but not the variances, indicating a floating screening cut point. For analyst 2, there were significant differences between the means for day, plate, and their interaction and the variances, indicating a dynamic cut point. Due to practical limitations of using a dynamic cut point, the validation study continued using analyst 1, thereby applying a floating cut point that was calculated as 1,066.6 RLUs (see Table 2, second iteration). The correction factor for the screening cut point for analyst 1 was estimated to be 128.6 RLUs, and this was applied to subsequent assays.

An analysis of the specificity cut-point data revealed a normal distribution and one outlier, which was excluded. The fixed specificity cut point was calculated to be 93% inhibition (Figure 1). Statistically significant differences were evident between the means for analyst, day, plate, analyst by day, day by plate, and analyst by day by plate interactions ($p < 0.001$).

Sensitivity

Assay sensitivity analysis was determined from data generated by analyst 1 only and was calculated as 356 ng/mL (see Table S2).

Controls

The NC samples were below the cut point, the LPC samples above the cut point, and the high positive control (HPC) samples at the high end of the dynamic range for both intra-assay and inter-assay analyses,

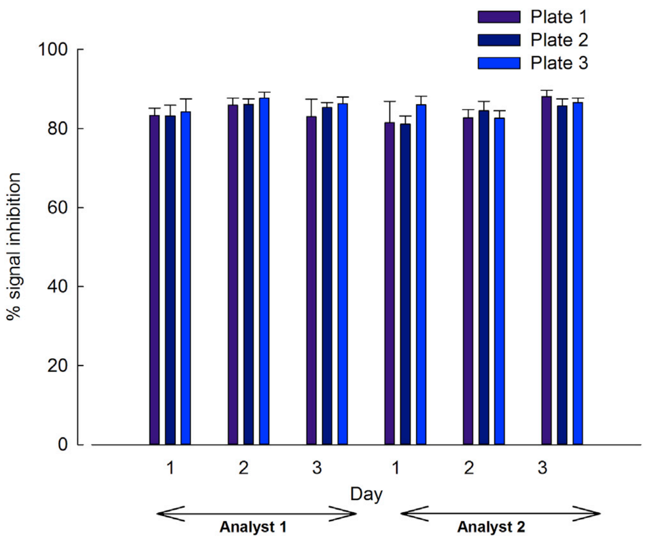

Figure 1. Specificity Cut Point

To establish the specificity cut point, we pre-incubated 51 individual control serum samples with TP at a concentration of 12,500 ng/mL and analyzed them in duplicate by two analysts over three plates on 3 days to assess % signal inhibition. Significant differences were observed between the means for analyst, day, plate, analyst × day, day × plate, and analyst × day × plate interactions ($p < 0.001$). Data are expressed as mean % signal inhibition ± SD.

and are therefore considered suitable. Controls pre-incubated in the presence of 12,500 ng/mL TP demonstrated RLUs below the cut point in both intra-assay and inter-assay analyses, with inhibitions ranging between 80.6% and 98.8% (Table 3).

Assay drift was not observed, as indicated by a mean difference in response readings of the control samples at the beginning and end of the assay plate being within ±30%, when compared with each other (data not shown).

Drug Tolerance

The drug tolerance of the analytical method was determined at 156 ng/mL (Figure 2).

Selectivity

All low and high spiked samples were above the cut point without TP and below the cut point with TP. Inhibitions at the confirmatory drug concentration (12,500 ng/mL) were observed for the high spiked patient and control samples (Figure 3). Matrix effects with regard to the therapeutic enzyme and disease state matrix are therefore not considered significant.

Prozone

The instrument response readings remained above the assay cut point; therefore, prozone effects were not observed up to a serum anti-TP antibody concentration of 25,900 ng/mL, 2.59-fold higher than the HPC (data not shown).

Stability

Anti-TP antibodies were stable up to 24 hr at room temperature and for up to five cycles of freeze-thaw at −70°C (Figure 4). The precision (% coefficient of variation [CV]) of the instrument responses was ≤20%.

Evaluation of Serum Samples from Treated Patients

Serum samples from three patients were analyzed before treatment and at different time points during treatment (Table 4). The mean instrument responses for all pre-treatment samples were below the assay cut point. For patient 1, one sample after 9 months of treatment was above the cut point. For patient 2, all samples during the treatment phase were above the cut point. For patient 3, one sample after 5 months of treatment was above the cut point. All positive samples from patient 2 were found to be specific in the confirmatory assay. Positive samples for patients 1 and 3 were confirmed as non-specific antibodies because the inhibition was below the specificity point of 93%.

DISCUSSION

Autologous erythrocyte-mediated enzyme replacement is employed as a strategy for preventing or minimizing the development of immune reactions against therapeutic enzymes. Our experience includes the treatment of a patient with adenosine deaminase deficiency with erythrocyte encapsulated adenosine deaminase and the administration of EETP to five patients with MNGIE under a compassionate use program.[5,6,14–17] A recombinant *E. coli* source of GMP TP has been developed to support a clinical trial of EETP. Although erythrocyte encapsulation would be predicted to reduce the immunogenicity of the enzyme, an intravascular release of TP from damaged erythrocytes is likely to evoke an immunogenic reaction against a protein of bacterial origin. The evaluation of the immunogenicity of therapeutic enzymes is an important aspect of clinical development because the formation of anti-enzyme antibodies can negatively influence the efficacy and safety of the proposed treatment.

In this study, we validated a method for the detection of anti-TP antibodies in the serum of patients treated with EETP according to published recommendations for the design and optimization of immunoassays for the detection of host antibodies against therapeutic proteins.[3,4,18–21] To minimize the false-positive rate and to increase specificity, we adopted a two-step analysis: a screening assay for the identification of anti-TP-positive patient samples, followed by an assay for confirming the presence of anti-TP antibodies. Due to having the potential to detect all antibody isotypes and classes produced in an immune response, an electrochemiluminescent bridging immunoassay platform was selected. Fifty-one individual control serum samples were used to determine the 95% confidence interval used as the cut-point factor. The cut-point factor was added to the mean signal for the pooled NC serum on each plate to establish the cut point. In the second analysis step, a confirmation assay was developed to confirm the specificity of putatively positive samples identified in the screening assay. In this approach, PC samples were pre-incubated with and without a high concentration of TP to inhibit the assay signal

Table 3. Intra-assay and Inter-assay Analysis of Control Samples with and without Pre-incubation with TP

Control Sample	Intra-assay			Inter-assay		
	RLUs (Mean ± SD)	CV (%)	% Inhibition	RLUs (Mean ± SD)	CV (%)	% Inhibition
NC	865 ± 126	14.5	–	483 ± 209	43.3	–
NC+TP	ND	ND	ND	87 ± 10	11.0	80.6
LPC	1,505 ± 167	11.1	–	919 ± 373	40.6	–
LPC+TP	151 ± 8	5.3	90.0	103 ± 27	25.8	88.9
HPC	18,111 ± 181	1.0	–	12,680 ± 3,873	30.5	–
HPC+TP	265 ± 3	1.0	98.5	158 ± 59	37.4	98.8

ND, not determined; TP, thymidine phosphorylase.

beyond the cut-point value; inhibition above the cut point confirmed the presence of anti-TP antibodies. Ideally, cut-point assessments should be conducted using disease state serum samples; however, for rare diseases, obtaining a sufficient number of patient samples is challenging. To address possible differences between control and diseases matrices, assay selectivity testing was assessed in patient and NC matrix samples. The bioanalytical guidelines of the European Medicines Agency (EMA) and US Food and Drug Administration (FDA) recommend the testing of at least 10 individual sources of sample matrix; however, because of the rarity of MNGIE, only seven patient matrix samples were available for testing.[22,23] The mean instrument responses between the patient and NC matrix samples nevertheless were not significantly different, therefore demonstrating the absence of disease matrix effects. Testing a larger number of samples will be contemplated during the clinical trial when more patients will be available.

The assay provided an adequate sensitivity of 356 ng/mL of polyclonal antibodies in serum; this is in the accepted range of 250–500 ng/mL in serum for antibody assays in clinical trials.[24] Drug tolerance was 156 ng/mL; in patient compassionate use studies, plasma levels of free TP are undetectable, and therefore assay interference by free TP is considered negligible.

No specific anti-TP antibodies were detected in patients 1 and 3, determined using the confirmatory assay. However, in patient 2, positive anti-TP antibodies were detected after 8 months of treatment (after nine administrations of EETP) onward. The development of anti-TP antibodies does not necessarily predict the development of adverse events in patients, but could potentially impact on the efficacy of TP by inhibiting the pharmacological activity of the enzyme through the formation of immune complexes. Another clinical consequence of antibody formation is cross-reactivity with an endogenous protein, which performs a key physiological function. The development of specific anti-TP antibodies in patient 2 did not raise any specific concerns with regard to the efficacy of encapsulated TP, because depletion of the plasma metabolites improved over the 5.5 years of administration, and clinical improvements were also recorded.[15] Nevertheless, heterogeneity in patient antibody responses are often observed, and thus sufficient data should be compiled during clinical development to characterize antibody response variability. Guidelines of the FDA and EMA recommend that specific antibody responses are further analyzed for neutralizing capacity.[3,4,25] Neutralizing antibody assay validation was not included in this study, and although we anticipate that it is unlikely that neutralizing antibodies will be formed due to the encapsulation of TP in the erythrocyte, a relevant assay will be validated during clinical development and prior to marketing authorization applications. Pre-clinical studies with EETP demonstrated specific anti-TP antibodies in 2 of 18 treated dogs and 19 of 60 treated BALB/c mice.[7] The development of specific antibodies against TP is not a surprising observation because senescent erythrocytes are naturally sequestered from the vascular compartment by macrophages of the monocyte-macrophage system, which is able to present antigens to T lymphocytes. We have previously shown that humoral responses can be elicited by the administration of erythrocyte encapsulated antigens to BALB/c mice.[26] One of the advantages of employing the autologous erythrocyte is that the development of antibodies against the carrier is unlikely, and indeed this has not been encountered in 25 years of clinical experience.

To conclude, this assay has appropriate performance characteristics and is considered suitable for the detection of anti-TP antibodies in human serum. Further assay refinement will be implemented during clinical development to include the validation of a neutralizing antibody assay and detection of IgE antibodies.

MATERIALS AND METHODS

This validation study was designed to adhere to recommendations for the validation of immunoassays used for detection of host antibodies against biotechnology products according to FDA and EMA immunogenicity guidelines and in compliance with Good Laboratory Practice (GLP) standards.[3,4,18–21,24]

Reagents

All reagents were supplied by Meso Scale Discovery, UK, unless otherwise stated. The wash buffer was PBS with 0.05% Tween 20 (Sigma Chemical Company, UK). Blocker A solution consisted of 5% (w/v) blocker A in phosphate buffer; the assay buffer was 1 vol of 5% blocker A solution and 4 vol of wash buffer, and the Read buffer (4×) was diluted 1:2 with ultra-high-purity-grade water.

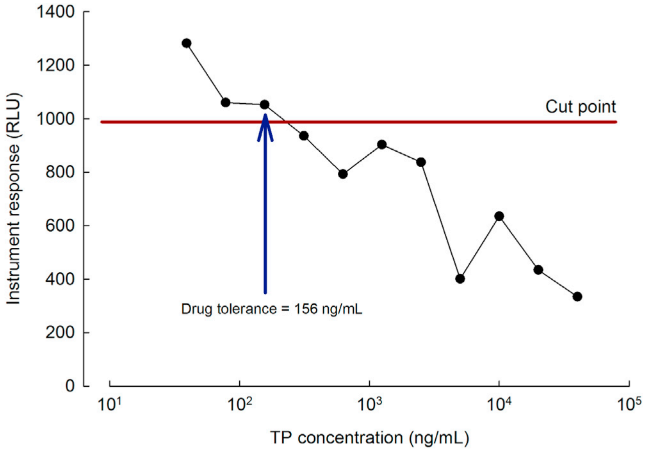

Figure 2. Assay Tolerance to Free TP

Instrument response as function of TP concentration. The LPC was spiked with TP over a concentration range of 39.1–40,000 ng/mL and incubated for 1 hr before analysis. The blue arrow indicates the assay drug tolerance.

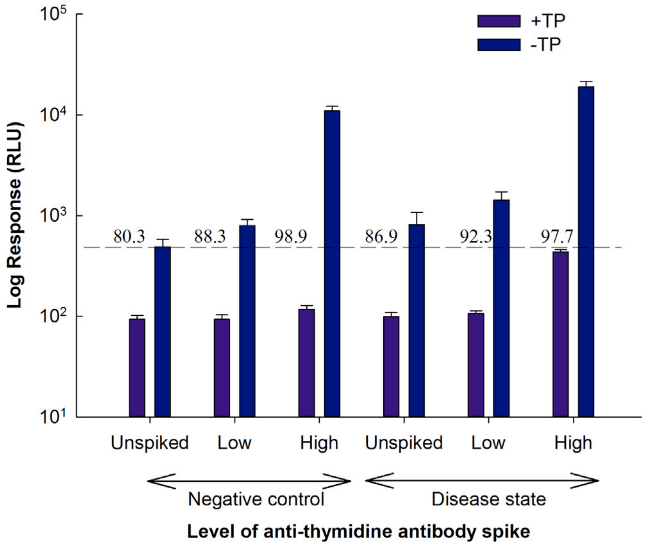

Figure 3. Assay Selectivity

Individual control (n = 10) and disease (n = 7) serum samples were unspiked or spiked with anti-TP antibodies at low (400 ng/mL) and high (10,000 ng/mL) concentrations. Two aliquots of each sample were prepared and incubated for 1 hr, one aliquot with buffer and the other aliquot with free TP (12,500 ng/mL). Dotted line represents assay cut point. Data are expressed as log mean RLUs ± SD.

Recombinant *E. coli* (TP, 13 mg/mL) produced by the methodology employed for the manufacture of clinical GMP material was employed for the development and validation of this immunoassay (Diatheva, Italy). A 12,500-ng/mL working solution of TP was prepared by dilution in assay buffer. Biotinylated and sulfo-TAG TP conjugates were prepared as the capture and detection antigens, respectively, as described previously and were used to formulate a conjugate mastermix complex working solution containing 300 ng/mL biotin and 300 ng/mL sulfo-TAG in assay buffer.[27]

Negative and Positive Human Serum Controls

An NC human serum pool was prepared from 15 individual human samples that had been screened against a positive control calibration curve for the presence of anti-TP antibodies and stored at −20°C until required. Positive human serum controls (PC) were prepared from affinity-purified rabbit anti-TP antibody (0.518 mg/mL; custom produced by Open Biosystems, Huntsville, AL, USA) diluted with NC sera to produce the low PC just above the cut point (low positive control [LPC], 400 ng/mL) and a high PC giving approximately 75% of the maximum signal (HPC, 10,000 ng/mL). Prior to analysis, the NC and PC samples were diluted 1:10 with assay buffer.

Samples from Patients with MNGIE

To ascertain that the normal matrix was representative of the disease state matrix, we screened and analyzed 7 individual treatment-naive disease state human serum samples alongside 10 individual NC samples.

Serum samples from three patients with a confirmed diagnosis of MNGIE who had received two to four weekly infusions of EETP (3.9–108 U/kg body weight) were collected pre-treatment and at a number of time points after therapy initiation. Samples were stored at −80°C in a temperature-monitored freezer until sample analysis, by a two-tiered process, consisting of a screening assay to identify samples positive for anti-TP antibodies, followed by a confirmatory assay to establish whether the antibodies were specific to TP. NC, LPC, and HPC samples were included in each assay run. Approval for the study was obtained from the National Research Ethics Service Committee. Patient informed consent was obtained prior to the start of treatment.

Assay Procedure

Assays were performed using an electrochemiluminescent bridging immunoassay. In brief, NC, PCs, and test samples were diluted in assay buffer with and without TP for 1 hr at room temperature, after which 75 μL was added to wells of a polypropylene 96-well plate (Fisher Scientific, UK) followed by 75 μL of conjugate mastermix. The plates were covered and incubated at room temperature for 2 hr with shaking at 800 rpm. Following this, 350 μL of blocker A solution was added to the appropriate wells of a streptavidin gold plate, which was then covered and incubated at room temperature for 2 hr with shaking at 800 rpm. The streptavidin plate was then washed three times with 350 μL of wash buffer per well using a plate washer; the last wash was aspirated and the plate blotted dry by inversion over absorbent paper. Two 50-μL aliquots from each well of the polypropylene 96-well plate were transferred to corresponding duplicate wells in the streptavidin plate, which was then covered and incubated at room temperature for 1 hr, with shaking at 800 rpm. This was followed by three washes with 350 μL of wash buffer per well using a plate washer; the last wash was aspirated and the plate blotted dry by inversion over absorbent paper. Finally, 150 μL of Read buffer (2×)

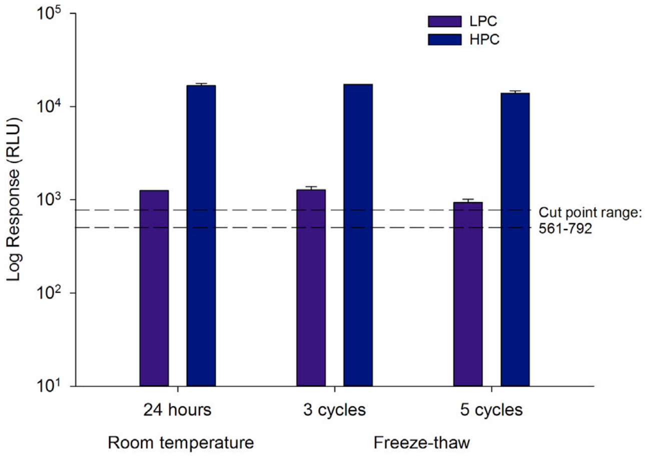

Figure 4. Stability of Anti-TP Antibody after 24 hr at Room Temperature and after Repeated Freeze-Thaw Cycles

The dashed lines represent the assay cut-point range. Data are expressed as log mean RLUs ± SD.

was added to each well the plate read on an MSD Sector Imager 6000 within 10 min.

Method Validation Parameters

Reagent Optimization

Design Expert was used to optimize the concentrations of biotinylated TP and sulfo-TAG TP.

PC Standard Curve for Assay Sensitivity Determination

PC calibration curves were prepared from working standards (n = 2, in duplicate) and processed using a four-parameter logistical algorithm; this fitting routine was applied throughout the determination of screening and specificity cut point and assay sensitivity.

Screening and Specificity Cut Point

The screening cut point was assessed to determine the threshold for identifying samples as negative or potentially positive (equal or above the cut point) for the presence of anti-TP antibodies. The methodology applied was that of Shankar et al.,[18] where the purpose was to determine the type of cut point required (floating, fixed, or dynamic), calculate the cut-point value, and determine the specificity (confirmation) cut point. Fifty-one individual human serum samples were measured in duplicate, by two analysts, over three plates, on 3 days.

The specificity cut point assay is employed to determine whether samples identified as potentially reactive in the screening assay are positive or negative for anti-TP antibodies. The same source of serum samples that were employed in the screening cut-point assay were pre-incubated with TP at a concentration of 12,500 ng/mL, this being 10 times the lowest concentration that was observed to fall below the screening cut point during assay development. Each assay run included a PC standard curve, NC, LPC, and HPC samples, with and without TP.

Table 4. Screening Analysis and Confirmatory Assay of Positive Patient Samples

Patient ID	Treatment (Months)	Screening Assay (-TP)	Confirmatory Assay	
			% Inhibition	Specificity
1	pre-treatment	negative	–	–
	9	positive	73.7	non-specific
	15	negative	–	–
	21	negative	–	–
	28	negative	–	–
2	pre-treatment	negative	–	–
	8	positive	95.5	specific
	16	positive	98.8	specific
	22	positive	97.3	specific
	28	positive	98.1	specific
	35	positive	98.5	specific
	41	positive	98.8	specific
	49	positive	99.1	specific
	60	positive	99.0	specific
	73	positive	97.6	specific
3	pre-treatment	negative	–	
	6	positive	75.6	non-specific

TP, thymidine phosphorylase.

Assay Sensitivity

The sensitivity of the assay is defined by the lowest concentration at which a PC antibody preparation consistently provides a positive signal in the assay. This was calculated as the mean concentration obtained by interpolation of the plate-specific cut-point value against the PC curve on each of the 18 assay runs described above and then determining the lowest concentration that is measured as positive 95% of the time. Instrument responses, RLUs for the PC samples, were assessed according to their relation to the cut point.

PC and NC Sample Suitability

Intra-assay precision was determined by the replicate analysis of NC (four independent preparations of NC, three independent preparations of LPC, and three independent preparations of the HPC samples in one assay run). An additional set of control samples pre-incubated with 12,500 ng/mL TP was also analyzed. Inter-assay precision was determined from the replicate analysis of 4 independent preparations of NC, 2 independent preparations of LPC, and 2 independent preparations of HPC samples, with and without TP on 15 occasions spanning 4 different days, by 2 analysts. Assay drift was assessed by analyzing control samples (+ pre-incubation with TP) in the first and last columns of the assay plate.

Drug Tolerance

The tolerance of the assay to free TP was assessed by pre-incubation of the LPC for 1 hr in TP over the final concentration range of 39.1–40,000 ng/mL. A sample without TP was also analyzed.

Selectivity

Assay selectivity was assessed to determine whether the assay was affected by the disease state matrix or by the potential existence of therapeutic TP in serum samples. Individual control (n = 10) and disease (n = 7) serum samples were unspiked and spiked with anti-TP antibodies at low (400 ng/mL) and high (10,000 ng/mL) concentrations. Two aliquots of sample were prepared and incubated for 1 hr, one aliquot with buffer and the other aliquot with assay buffer containing free TP (12,500 ng/mL). The samples were distributed over four assay runs. The percentage inhibition of signal in the presence of free TP was calculated using the formula described in Data Handling and Statistics.

Prozone

Assay prozone caused by high anti-TP antibody levels was investigated by serial dilution of a high spiked sample (containing anti-TP antibodies at a concentration of 25,900 ng/mL) with assay buffer.

Stability

The effect of anticipated sample handling conditions on assay performance was evaluated, specifically bench top storage at room temperature (nominally 22°C) and repeated freeze-thaw cycles. Room temperature effects were assessed by thawing one set of PC samples for approximately 24 hr (expected maximum duration that samples would be left thawed) and an additional set for baseline assessment, just prior to analysis (n = 3, in duplicate). The effect of repeated freeze-thaw cycles on the stability of anti-TP antibodies was assessed by subjecting PC samples to three and five freeze-thaw cycles, with each cycle consisting of a minimum of 2 hr at room temperature, followed by storage at −70°C for at least 12 hr (n = 3, in duplicate). An additional set of PC samples for baseline assessment was thawed prior to analysis. Stability was verified if the mean precision (% CV) and mean percent difference from the baseline responses were $\leq$20%.

Data Handling and Statistics

Instrument responses are reported as mean values of RLU. All data acquisition, processing, and evaluations were performed using the Watson Laboratory Information Management System version 7.2, Microsoft Excel, and Meso Scale Discovery Workbench version 3.0.185. Data for cut-point calculation were analyzed using SAS Version 9.1.3.

Assay cut-point evaluation was performed using the statistical methodology described by Shankar et al.[18] Measurements for each of the 51 human serum samples (n = 18, in duplicate) were averaged and tested for normality using the Shapiro-Wilk's test;[28] logarithmic (base 10) or square root transformation was applied to non-normally distributed data. An assessment for outliers was made using the Studentized Deleted Residuals whereby residues <−3 or >3 SDs were excluded. Once outliers were removed, data were reassessed for normality; the validation cut point was defined as the 95% quantile for non-normally distributed data, or the mean + 1.645*SD for normally distributed data.

To assess the type of screening cut point to apply, we applied an ANOVA method to assess for any analyst, plate, and day differences on either the untransformed or transformed data, depending on the outcome of the Shapiro-Wilk's test above. Analyst, plate, day, and their interactions were set as fixed factors, whereas subject was included as a random effect. Levene's test for homogeneity of variance was performed.[29] A fixed screening cut point was indicated if there were no differences or variances, whereas a floating cut point was reported if there were differences between means only; otherwise a dynamic screening cut point was required.[18] The correction factor was calculated as the validation cut point minus the mean of the NC values from the validation runs. The screening cut point was defined as either the validation cut point or the mean of NC values from the in-study run + correction factor, depending on whether the means and variances between runs were similar.

The fixed specificity cut point was calculated using the method of Shankar et al.[18] For each sample, the percentage inhibition of signal in the presence of free TP was calculated as follows:

$$Signal\ inhibition(\%) = 100 \times \left[1 - \left(\frac{drug\ inhibited\ sample}{unihibited\ sample}\right)\right].$$

Data were assessed for outliers and normal distribution and treated accordingly, as described above. For normally distributed data, the fixed specificity cut point was calculated as mean % inhibition + 3.09 × SD. For data not normalized by transformations, the specificity cut point was calculated as medium + 99% quantile. ANOVA techniques were applied to assess for analyst, plate, and day differences.

In the event of differences between analysts for either inhibited or uninhibited samples, the sensitivity analysis was performed separately for each analyst. Each dilution curve was analyzed using a four-parameter model. For a floating cut point, separate curves were analyzed for each plate, whereas for a fixed cut point the data were combined from all plates. The screening assay cut point determined to be appropriate for the method was back-calculated onto the standard curve for each plate to obtain the log concentrations of the screening cut points. These were averaged across all plates and a 95% confidence interval obtained for the overall mean on the log scale. The back-transformed upper 95% confidence interval was calculated, which was defined as the sensitivity of the assay.

SUPPLEMENTAL INFORMATION

Supplemental Information includes one figure and two tables and can be found with this article online at https://doi.org/10.1016/j.omtm.2018.08.007.

AUTHOR CONTRIBUTIONS

M.L., D.P., C.G., J.H., M.S.-M., and B.E.B. contributed to the design and implementation of the research, to the analysis of the results, and to the writing of the manuscript.

CONFLICTS OF INTEREST

The authors have no conflicts of interest.

ACKNOWLEDGMENTS

This work was supported by a Medical Research Council grant (reference MR/K025406/1). We highly appreciate the contributions of Dr. Moshe Baru (Orphan Technologies).

REFERENCES

1. Baldo, B.A. (2015). Enzymes approved for human therapy: indications, mechanisms and adverse effects. BioDrugs *29*, 31–55.
2. Kishnani, P.S., Dickson, P.I., Muldowney, L., Lee, J.J., Rosenberg, A., Abichandani, R., Bluestone, J.A., Burton, B.K., Dewey, M., Freitas, A., et al. (2016). Immune response to enzyme replacement therapies in lysosomal storage diseases and the role of immune tolerance induction. Mol. Genet. Metab. *117*, 66–83.
3. U.S. Food and Drug Administration. (2014). Guidance for industry: immunogenicity assessment for therapeutic protein products. Report of the U.S. Department of Health and Human Services, Food and Drug Administration, Center for Drug Evaluation and Research (CDER), and Center for Biologics Evaluation and Research (CBER). August 2014. https://www.fda.gov/downloads/drugs/guidances/ucm338856.pdf.
4. European Medicines Agency. (2017). Guideline on immunogenicity assessment of therapeutic proteins. May 18, 2017. EMEA/CHMP/BMWP/14327/2006 Rev 1. http://www.ema.europa.eu/docs/en_GB/document_library/Scientific_guideline/2017/06/WC500228861.pdf.
5. Godfrin, Y., Horand, F., Franco, R., Dufour, E., Kosenko, E., Bax, B.E., Banz, A., Skorokhod, O.A., Lanao, J.M., Vitvitsky, V., et al. (2012). International seminar on the red blood cells as vehicles for drugs. Expert Opin. Biol. Ther. *12*, 127–133.
6. Godfrin, Y., and Bax, B.E. (2012). Enzyme bioreactors as drugs. Drugs Future *37*, 263–272.
7. Levene, M., Coleman, D.G., Kilpatrick, H.C., Fairbanks, L.D., Gangadharan, B., Gasson, C., and Bax, B.E. (2013). Preclinical toxicity evaluation of erythrocyte-encapsulated thymidine phosphorylase in BALB/c mice and beagle dogs: an enzyme-replacement therapy for mitochondrial neurogastrointestinal encephalomyopathy. Toxicol. Sci. *131*, 311–324.
8. Hirano, M., Silvestri, G., Blake, D.M., Lombes, A., Minetti, C., Bonilla, E., Hays, A.P., Lovelace, R.E., Butler, I., Bertorini, T.E., et al. (1994). Mitochondrial neurogastrointestinal encephalomyopathy (MNGIE): clinical, biochemical, and genetic features of an autosomal recessive mitochondrial disorder. Neurology *44*, 721–727.
9. Nishino, I., Spinazzola, A., and Hirano, M. (1999). Thymidine phosphorylase gene mutations in MNGIE, a human mitochondrial disorder. Science *283*, 689–692.
10. Martí, R., Nishigaki, Y., and Hirano, M. (2003). Elevated plasma deoxyuridine in patients with thymidine phosphorylase deficiency. Biochem. Biophys. Res. Commun. *303*, 14–18.
11. Nishigaki, Y., Martí, R., Copeland, W.C., and Hirano, M. (2003). Site-specific somatic mitochondrial DNA point mutations in patients with thymidine phosphorylase deficiency. J. Clin. Invest. *111*, 1913–1921.
12. Valentino, M.L., Martí, R., Tadesse, S., López, L.C., Manes, J.L., Lyzak, J., Hahn, A., Carelli, V., and Hirano, M. (2007). Thymidine and deoxyuridine accumulate in tissues of patients with mitochondrial neurogastrointestinal encephalomyopathy (MNGIE). FEBS Lett. *581*, 3410–3414.
13. Garone, C., Tadesse, S., and Hirano, M. (2011). Clinical and genetic spectrum of mitochondrial neurogastrointestinal encephalomyopathy. Brain *134*, 3326–3332.
14. Moran, N.F., Bain, M.D., Muqit, M.M., and Bax, B.E. (2008). Carrier erythrocyte entrapped thymidine phosphorylase therapy for MNGIE. Neurology *71*, 686–688.
15. Bax, B.E., Bain, M.D., Scarpelli, M., Filosto, M., Tonin, P., and Moran, N. (2013). Clinical and biochemical improvements in a patient with MNGIE following enzyme replacement. Neurology *81*, 1269–1271.
16. Bax, B.E., Bain, M.D., Fairbanks, L.D., Webster, A.D.B., and Chalmers, R.A. (2000). In vitro and in vivo studies with human carrier erythrocytes loaded with polyethylene glycol-conjugated and native adenosine deaminase. Br. J. Haematol. *109*, 549–554.
17. Bax, B.E., Bain, M.D., Fairbanks, L.D., Webster, A.D., Ind, P.W., Hershfield, M.S., and Chalmers, R.A. (2007). A 9-yr evaluation of carrier erythrocyte encapsulated adenosine deaminase (ADA) therapy in a patient with adult-type ADA deficiency. Eur. J. Haematol. *79*, 338–348.
18. Shankar, G., Devanarayan, V., Amaravadi, L., Barrett, Y.C., Bowsher, R., Finco-Kent, D., Fiscella, M., Gorovits, B., Kirschner, S., Moxness, M., et al. (2008). Recommendations for the validation of immunoassays used for detection of host antibodies against biotechnology products. J. Pharm. Biomed. Anal. *48*, 1267–1281.
19. Secretary of State for Health. (2004). The good laboratory practice (codification amendments etc.) regulations 2004 (statutory instrument 1999 no. 3106, as amended by statutory instrument 2004 no. 994). Report of the Department of Health. April 27, 2004. http://www.legislation.gov.uk/uksi/2004/994/pdfs/uksi_20040994_en.pdf
20. Organisation for Economic Co-operation and Development. (1998). OECD series on principles of good laboratory practice and compliance monitoring: OECD principles of good laboratory practice (as revised in 1997). ENV/MC/CHEM (98)17. http://www.oecd.org/officialdocuments/publicdisplaydocumentpdf/?cote=env/mc/chem(98)17&doclanguage=en.
21. Directive 2004/10/EC of the European Parliament and of the Council (Official Journal No. L 50/44). Report of the European Parliament and the Council of the European Union. Official Journal of the European Union. February 11, 2004. https://www.bfr.bund.de/cm/349/directive_2004_10_ec.pdf.
22. U.S. Food and Drug Administration. (2018). Bioanalytical method validation: guidance for industry. Report of the U.S. Department of Health and Human Services, Food and Drug Administration, Center for Drug Evaluation and Research (CDER), and Center for Biologics Evaluation and Research (CBER). May 2018. https://www.fda.gov/downloads/drugs/guidances/ucm070107.pdf.
23. European Medicines Agency. (2011). Guideline on bioanalytical method validation. EMEA/CHMP/EWP/192217/2009 Rev. 1 Corr. Report of the Committee for Medicinal Products for Human Use. July 21, 2011. http://www.ema.europa.eu/docs/en_GB/document_library/Scientific_guideline/2011/08/WC500109686.pdf.
24. Mire-Sluis, A.R., Barrett, Y.C., Devanarayan, V., Koren, E., Liu, H., Maia, M., Parish, T., Scott, G., Shankar, G., Shores, E., et al. (2004). Recommendations for the design and optimization of immunoassays used in the detection of host antibodies against biotechnology products. J. Immunol. Methods *289*, 1–16.
25. Gupta, S., Devanarayan, V., Finco, D., Gunn, G.R., 3rd, Kirshner, S., Richards, S., Rup, B., Song, A., and Subramanyam, M. (2011). Recommendations for the validation of cell-based assays used for the detection of neutralizing antibody immune responses elicited against biological therapeutics. J. Pharm. Biomed. Anal. *55*, 878–888.
26. Murray, A.M., Pearson, I.F., Fairbanks, L.D., Chalmers, R.A., Bain, M.D., and Bax, B.E. (2006). The mouse immune response to carrier erythrocyte entrapped antigens. Vaccine *24*, 6129–6139.
27. Gasson, C., Levene, M., and Bax, B.E. (2013). The development and validation of an immunoassay for the measurement of anti-thymidine phosphorylase antibodies in mouse and dog sera. J. Pharm. Biomed. Anal. *72*, 16–24.
28. Shapiro, S.S., and Wilk, M.B. (1965). An analysis of variance test for normality (complete samples). Biometrika *52*, 591–611.
29. Levene, H. (1960). Robust tests for equality of variances. In Contributions to Probability and Statistics, I. Olkin, S.G. Ghurye, W. Hoeffding, W.G. Madow, and H.B. Manneds, eds. (Stanford University Press), pp. 278–292.

OMTM, Volume *11*

Supplemental Information

Validation of an Immunoassay for Anti-thymidine Phosphorylase Antibodies in Patients with MNGIE Treated with Enzyme Replacement Therapy

Michelle Levene, Dario Pacitti, Charlotte Gasson, Jamie Hall, Marcia Sellos-Moura, and Bridget E. Bax

Supplemental Figure 1. Screening cut-point. To establish the screening cut-point, 51 individual control serum samples were analysed in duplicate by two analysts over three plates on three days. Significant differences were observed between means for analyst, day, plate, analyst*plate, analyst*day and analyst*day*plate interactions (p< 0.001) and variances (p<0.001). Data is expressed as mean RLU ± SD.

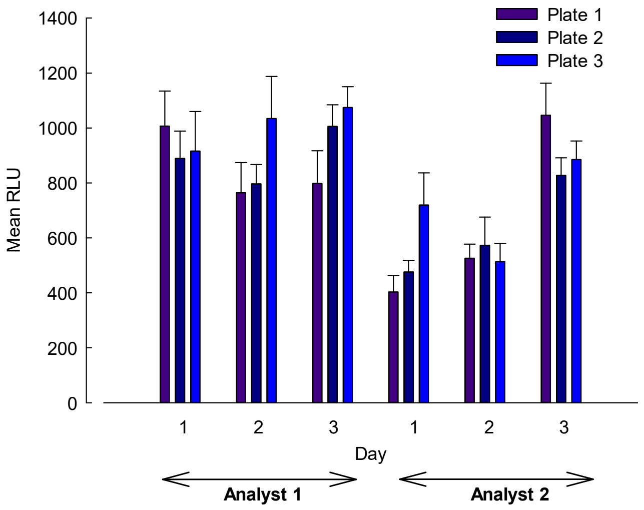

Supplemental Table 1. Comparison of instrument responses to negative control and disease state matrices.

Serum sample	Instrument response (RLU[a])	
	**Mean ± SD**	**CV (%)**
Disease (n=5)	1169 ± 388	33.2
Healthy control (n=10)	1301 ± 141	10.9

[a] RLU; relative light unit

Supplemental Table 2. Sensitivity analysis for Analyst 1 for each of 9 plates.

Plate	Mean RLU[a] Negative control	Screening cut point[b] (RLU)	Log sensitivity level
1	1037.0	1165.6	1.171
2	981.0	1109.6	2.233
3	905.0	1033.6	2.422
4	920.5	1049.1	3.114
5	818.0	946.6	2.472
6	810.5	939.1	2.243
7	987.5	1116.1	1.803
8	1107.0	1235.6	2.119
9	875.5	1004.1	2.431
**Log mean sensitivity level + SD**			2.223 ± 0.528
**Log mean sensitivity Upper 95% CI[c]**			2.551
**Mean sensitivity Upper 95% CI**			355.53

[a] RLU; relative light unit

[b]Screening cut point = Negative control mean + Correction Factor (128.6)

[c] Upper log mean sensitivity =Mean + (SD/√n)*t
